# Time-Restricted Feeding Regulates Circadian Rhythm of Murine Uterine Clock

**DOI:** 10.1093/cdn/nzab064

**Published:** 2021-04-09

**Authors:** Takashi Hosono, Masanori Ono, Takiko Daikoku, Michihiro Mieda, Satoshi Nomura, Kyosuke Kagami, Takashi Iizuka, Rieko Nakata, Tomoko Fujiwara, Hiroshi Fujiwara, Hitoshi Ando

**Affiliations:** Department of Obstetrics and Gynecology, Graduate School of Medical Science, Kanazawa University, Kanazawa, Japan; Department of Obstetrics and Gynecology, Graduate School of Medical Science, Kanazawa University, Kanazawa, Japan; Institute for Experimental Animals, Advanced Science Research Center, Graduate School of Medical Science, Kanazawa University, Kanazawa, Japan; Department of Integrative Neurophysiology, Graduate School of Medical Science, Kanazawa University, Kanazawa, Japan; Department of Obstetrics and Gynecology, Graduate School of Medical Science, Kanazawa University, Kanazawa, Japan; Department of Obstetrics and Gynecology, Graduate School of Medical Science, Kanazawa University, Kanazawa, Japan; Department of Obstetrics and Gynecology, Graduate School of Medical Science, Kanazawa University, Kanazawa, Japan; Department of Food Science and Nutrition, Nara Women's University, Nara, Japan; Department of Social Work and Life Design, Kyoto Notre Dame University, Kyoto, Japan; Department of Obstetrics and Gynecology, Graduate School of Medical Science, Kanazawa University, Kanazawa, Japan; Department of Cellular and Molecular Function Analysis, Graduate School of Medical Science, Kanazawa University, Kanazawa, Japan

**Keywords:** circadian rhythms, clock gene, dysmenorrhea, meal timing, uterine function

## Abstract

**Background:**

Skipping breakfast is associated with dysmenorrhea in young women. This suggests that the delay of food intake in the active phase impairs uterine functions by interfering with circadian rhythms.

**Objectives:**

To examine the relation between the delay of feeding and uterine circadian rhythms, we investigated the effects of the first meal occasion in the active phase on the uterine clock.

**Methods:**

Zeitgeber time (ZT) was defined as ZT0 (08:45) with lights on and ZT12 (20:45) with lights off. Young female mice (8 wk of age) were divided into 3 groups: group I (ad libitum consumption), group II (time-restricted feeding during ZT12–16, initial 4 h of the active period), and group III (time-restricted feeding during ZT20–24, last 4 h of the active period, a breakfast-skipping model). After 2 wk of dietary restriction, mice in each group were killed at 4-h intervals and the expression profiles of uterine clock genes, *Bmal1* (brain and muscle aryl hydrocarbon receptor nuclear translocator-like protein 1), *Per1* (period circadian clock 1), *Per2*, and *Cry1* (cryptochrome 1), were examined.

**Results:**

qPCR and western blot analyses demonstrated synchronized circadian clock gene expression within the uterus. Immunohistochemical analysis confirmed that BMAL1 protein expression was synchronized among the endometrium and myometrium. In groups I and II, mRNA expression of *Bmal1* was elevated after ZT12 at the start of the active phase. In contrast, *Bmal1* expression was elevated just after ZT20 in group III, showing that the uterine clock rhythm had shifted 8 h backward. The changes in BMAL1 protein expression were confirmed by western blot analysis.

**Conclusions:**

This study is the first to indicate that time-restricted feeding regulates a circadian rhythm of the uterine clock that is synchronized throughout the uterine body. These findings suggest that the uterine clock system is a new candidate to explain the etiology of breakfast skipping–induced uterine dysfunction.

## Teaser

This study indicates that time-restricted feeding regulates circadian clock of the murine uterus, suggesting the involvement of a uterine clock system in the relation between inadequate diets and uterine dysfunction.

## Introduction

Inadequate dieting and meal-skipping are currently important nutritional problems in young women (1–3). About 2 decades ago, we reported that breakfast skipping was significantly correlated with a high incidence of dysmenorrhea in Japanese female college students (4). Later, a similar correlation between breakfast skipping and dysmenorrhea was reported (5–8). Interestingly, Japanese students with breakfast skipping showed no decrease of BMI (4, 9). Consequently, considering that breakfast skipping is meal-skipping at the start of the active phase, we proposed that breakfast skipping impairs the reproductive functions by disrupting central and/or peripheral clock systems (9, 10). Using young female rats, we investigated the above concept and observed the supporting findings that the absence of feeding in active phases impairs ovarian functions (11).

A study showed that feeding during an inactive phase desynchronizes peripheral clocks, causing obesity and metabolic disorders in adult mice (12). The mouse model for breakfast skipping showed that the first meal occasion in the active phase regulates peripheral clock gene expression in the liver (13). In a circadian oscillator system, transcription factors, clock circadian regulator (CLOCK), and brain and muscle aryl hydrocarbon receptor nuclear translocator-like protein 1 (BMAL1), form a heterodimer and activate the transcription of clock-controlled genes and core clock genes, period circadian clock (*Per1–3*) and cryptochrome 1 and 2 (*Cry1* and *Cry2*). These *Per* and *Cry* proteins heterodimerize and repress their own transcription by interacting with CLOCK/BMAL1 complexes. This negative feedback loop creates a 24-h cycle in cells, which is usually synchronized within an organ (14). In humans, reduction of *BMAL1* gene expression in the endometrium was demonstrated in women with recurrent spontaneous abortion (15). This study also showed that the knockdown of *BMAL1* gene expression in decidual cells impaired their ability to regulate trophoblast invasion (15), suggesting that peripheral clock genes play important roles in human uterine functions. In rodents, circadian clock genes such as *Per1–3*, *Cry1* and *Cry2*, *Bmal1*, and *Clock* were shown to be expressed in the uterus (16, 17). Our preliminary experiments have demonstrated that uterus-specific *Bmal1*-deficient mice showed placental dysfunction, leading to the failed maintenance of pregnancy (M Ono et al., unpublished results, 2021).

Based on this background, in the present study, we examined the effects of the first meal occasion in the active phase on the uterine clock using young female mice to investigate the possibility that breakfast skipping affects a peripheral clock system in the uterus, which might explain the etiology of breakfast skipping–induced uterine dysfunction.

## Methods

### Animals

Seven-week-old female wild-type C57BL/6J mice (*n *= 88) were purchased from SLC Japan, and housed in stainless steel cages under a 12-h light/dark schedule. Mice were acclimatized for 1 wk, during which time a commercial experimental diet (21.9% protein, 55.3% carbohydrate, and 5.4% fat, 3.57 kcal/g; CRF-1; Oriental Yeast Co., Ltd) and water were available ad libitum. Mice were then allocated to various time-limited feeding groups. All experimental procedures and housing conditions were approved by the Animal Care and Use Committee of the Kanazawa University Animal Experiment Committee (Approval Number, AP-183942), and all animals were treated in accordance with the Institutional Guidelines for Experiments Using Animals. All studies involving animals are reported in accordance with the ARRIVE guidelines for reporting experiments involving animals (18).

### Time-restricted feeding protocol

The Zeitgeber time (ZT) was used to represent the experimental time, which was defined as ZT0 (08:45) with lights on and ZT12 (20:45) with lights off. After 1 wk of acclimation, the mice (*n *= 54) were divided into 3 groups: group I (consuming ad libitum), group II (time-restricted feeding during ZT12–16, initial 4 h of the active period), and group III (time-restricted feeding during ZT20–24, last 4 h of the active period). Group II represents young women who take breakfast without food intake during the night (nonactive phase), whereas group III represents breakfast skippers who take lunch or dinner without food intake during the night. We chose 8-h intervals between group II and group III to clearly detect the shift of circadian rhythms of clock genes. Each group (*n *= 18) was assigned to a different dietary schedule at the beginning of 8 wk of age. After 2 wk of time-restricted feeding, mice (*n *= 3) in each group were killed by cervical dislocation at 6 time points: ZT0, 4, 8, 12, 16, and 20, and bilateral uteri were resected. The uterine samples were frozen or fixed with paraformaldehyde (PFA) for subsequent western blot, PCR, or immunohistochemical analyses.

### RNA extraction and real-time qPCR

Uterine samples were divided into upper and lower portions and stored at −80°C. Extraction of total RNA and reverse transcription into cDNA were performed using the RNeasy Mini Kit (Qiagen) and the PrimeScript RT reagent Kit (Takara Bio Inc.), respectively. Gene expression was analyzed with the intercalator method using the real-time PCR system Mx3000p (Agilent Technologies Inc.) or by probe assay using the VIIA 7 Real-Time PCR system (Thermo Fisher Scientific). Expression of the target gene was normalized to expression of the endogenous control *Gapdh* (glyceraldehyde 3-phosphate dehydrogenase) in the intercalator method and *Rplp0* (ribosomal protein lateral stalk subunit P0) in the probe assay. Data were analyzed using the comparative threshold cycle method. Primer sequences and sets of specific primers and TaqMan probes are shown in **Table 1**.

**TABLE 1:** Primer sequences for RT-PCR and quantitative real-time PCR^1^

Gene	GenBank accession number	Assay ID
*Bmal1*	NM_007489.4	Mm00500226_m1
*Per1*	NM_011065.4	Mm00501813_m1
*Per2*	NM_011066.3	Mm00478113_m1
*Cry1*	NM_007771.3	Mm00514392_m1
*Krt7*	NM_033073.3	Mm00466676_m1
*Acta2*	NM_033073.3	Mm00466676_m1
*Rplp0*	NM_007475.5	Mm00725448_s1
**Gene**	**Forward primers**	**Reverse primers**
*Bmal1*	CCTAATTCTCAGGGCAGCAGAT	TCCAGTCTTGGCATCAATGAGT
*Per1*	GTGTCGTGATTAAATTAGTCAG	ACCACTCATGTCTGGGCC
*Per2*	GCGGATGCTCGTGGAATCTT	GCTCCTTCAGGGTCCTTATC
*Cry1*	TTGCCTGTTTCCTGACTCGT	GACAGCCACATCCAACTTCC
*Krt7*	CGCCGCTGAGTGTGGACATCG	CTGGCTGCTCTTGGCTGACTTCTG
*Acta2*	GAGGCACCACTGAACCCTA	CATCTCCAGAGTCCAGCACA
*Rplp0*	CTCTCGCTTTCTGGAGGGTG	TCAGTCTCCACAGACAATGCC
*Gapdh*	CATGGCCTTCCGTGTTCCT	CCTGCTTCACCACCTTCTTGA

1 *Acta2*, smooth muscle marker αSMA; *Bmal1*, brain and muscle aryl hydrocarbon receptor nuclear translocator-like protein 1; CK-7, cytokeratin 7; *Cry1*, cryptochrome 1; *Gapdh*, glyceraldehyde 3-phosphate dehydrogenase; *Krt7*, endometrial epithelial marker CK-7; *Per*, period circadian clock; *Rplp0*, ribosomal protein lateral stalk subunit P0; αSMA, α smooth muscle actin.

### Immunohistochemical staining

To analyze the protein abundance and localization of *Bmal1* expression in the uterus, immunohistochemical staining was performed. The uterine samples were fixed with 4% PFA in PBS, as previously described (19). The uterine tissues were embedded in optimal cutting temperature compound (Sakura Finetek Japan Co., Ltd). Tissue sections of 8-μm thickness were made using a cryostat, permeabilized with 0.5% Triton X-100 in PBS, and incubated at 4°C overnight with the first antibody (19, 20). For indirect immunofluorescence staining, after being incubated at 37°C for 2 h with Alexa 488-conjugated secondary antibody and 1 μg/mL Hoechst 33342, the sections were washed and mounted with Mowiol (Sigma-Aldrich) (19, 20). Immunohistochemical staining was performed using a standard avidin-biotin complex peroxidase method, as described previously (21). Antibodies used for immunostaining were as follows: rabbit anti-Bmal1 antibody (1:100; NB100-2288; Novus Biological), rabbit anti-cytokeratin-7 (CK-7) antibody (1:4000; ab181598; Abcam), and rabbit anti-α smooth muscle actin (αSMA) antibody (1:500; ab5694; Abcam). Horizontal images (×100) were captured using an epifluorescence microscope BZ-X710 (Keyence).

### Western blot analysis

To analyze the protein abundance of BMAL1 in the uterine samples, western blot analysis was performed. Protein lysates were extracted from frozen uterine samples with RIPA Buffer (Cell Signaling Technology Inc.). Each lysate was electrophoresed on a 10% SDS-PAGE gel and then transferred to a nitrocellulose membrane. The transferred membranes were incubated with primary antibody against BMAL1 (1:2000) overnight at 4°C. Secondary horseradish peroxidase–conjugated antibody was applied for 1 h at room temperature. β-Actin was used as a control. The blots were visualized with an enhanced chemiluminescence system using ECLTM Western Blotting Detection Reagents (GE Healthcare).

### Statistical analysis

The cyclic rhythms in mRNA and protein expression of clock genes and differences in mRNA expression at each ZT among groups I–III were analyzed by 1-factor ANOVA followed by the Dunnett test, and differences in mRNA expression of *Krt7* (endometrial epithelial marker CK-7), *Acta2* (smooth muscle marker αSMA), and clock genes between the endometrium and myometrium were analyzed by the paired *t* test using statistical software (SPSS version 25.0; IBM). The values of expression intensity of mRNA by qPCR are shown as the ratio per inner positive control gene. A *P* value <0.05 was considered significant.

## Results

### *Bmal1* was expressed in the murine uterus

To evaluate the circadian rhythm and synchronization of the uterine clock system, we initially assessed the reliability of detecting mRNA and protein of *Bmal1*, *Krt7*, and *Acta2*, by immunohistochemical, western blot, and RT-PCR analyses using 10-wk-old female mice (consuming ad libitum, *n *= 6).

**FIGURE 1:**
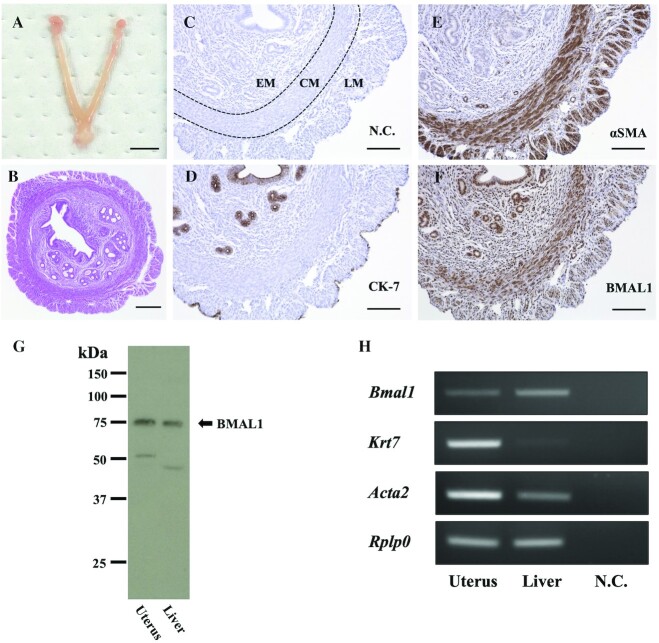
*Bmal1* expression in the murine uterus. (A) Bicornuate uterus. (B) Hematoxylin and eosin staining of a horizontal section of the uterus. (C) Negative control. (D) CK-7 was mainly expressed in the endometrium. (E) αSMA was expressed in both the inner circular and outer longitudinal muscle layers. (F) BMAL1 expression was observed in both the endometrium and myometrium. Bars show 500 µm (A), 200 µm (B), and 100 µm (C–F). (G) Western blot analysis detected specific bands at a molecular weight of 75 kDa in the uterus and liver, which correspond to BMAL1. (H) RT-PCR confirmed the mRNA expression of *Bmal1*, *Krt7*, and *Acta2* in the uterus. *Acta2*, smooth muscle marker αSMA; BMAL1, brain and muscle aryl hydrocarbon receptor nuclear translocator-like protein 1; CK-7, cytokeratin 7; CM, circular muscle; EM, endometrium; *Krt7*, endometrial epithelial marker CK-7; LM, longitudinal muscle; N.C., negative control; *Rplp0*, ribosomal protein lateral stalk subunit P0; αSMA, α smooth muscle actin.

Horizontal sections of the murine uterus (**Figure 1**A) were histologically examined by hematoxylin and eosin staining (Figure 1B) and immunohistochemical staining (Figure 1C–F). CK-7 was mainly expressed in the endometrium (Figure 1D), whereas αSMA was expressed in both the inner circular and outer longitudinal muscle layers (Figure 1E). BMAL1 expression was observed in both the endometrium and myometrium (Figure 1F). Western blot analysis detected specific bands at a molecular weight of ∼75 kDa in the uterus and liver, which correspond to BMAL1 protein (Figure 1G). RT-PCR confirmed the mRNA expression of *Bmal1*, *Krt7*, and *Acta2* in the uterus (Figure 1H).

### Uterine clock genes showed circadian rhythms

**FIGURE 2:**
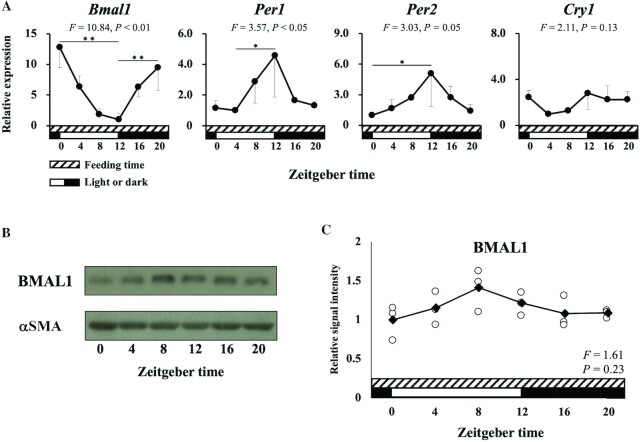
Circadian rhythms of uterine clock genes. By qPCR analyses, circadian expression profiles of clock genes in the murine uterus were examined. (A) Circadian rhythms of uterine clock genes in group I (ad libitum consumption). The mRNA expression of *Bmal1*, *Per1*, *Per2*, and *Cry1* in the uterus showed circadian cycles. Relative expression is presented as fold units per minimal values. The mRNA expression of *Bmal1* significantly rises after the start of the dark period (ZT12) and reaches a peak at the end of the dark period (ZT0). (B, C) Although nonsignificant, western blot analysis demonstrated circadian expression profiles of BMAL1 protein in the uterine tissues. The peak of protein expression was observed 8 h after that of mRNA expression. The cyclic changes in mRNA and protein expression of clock genes were analyzed by 1-factor ANOVA followed by the Dunnett test. **P < 0.01, *P < 0.05 between the groups at either end of the bar. αSMA, α-smooth muscle actin; *Bmal1*, brain and muscle aryl hydrocarbon receptor nuclear translocator-like protein 1; *Cry1*, cryptochrome 1; *Per*, period circadian clock; ZT, Zeitgeber time.

In group I (ad libitum consumption), the mRNA expression of peripheral clock genes (*Bmal1*, *Per1*, *Per2*, and *Cry1*) in the uterus showed circadian cycles (**Figure 2**A). For example, a circadian cycle of *Bmal1* showed that its mRNA expression rises after the start of the dark period (ZT12) and reaches a peak at the end of the dark period (ZT0). In accordance with mRNA expression, western blot analysis demonstrated a circadian cycle of BMAL1 protein expression in the uterine tissues and its higher expression at ZT8 (Figure 2B and C). Although nonsignificant, the peak of protein expression was observed 8 h after that of mRNA expression.

### Clock gene expression was synchronized in the uterus

**FIGURE 3:**
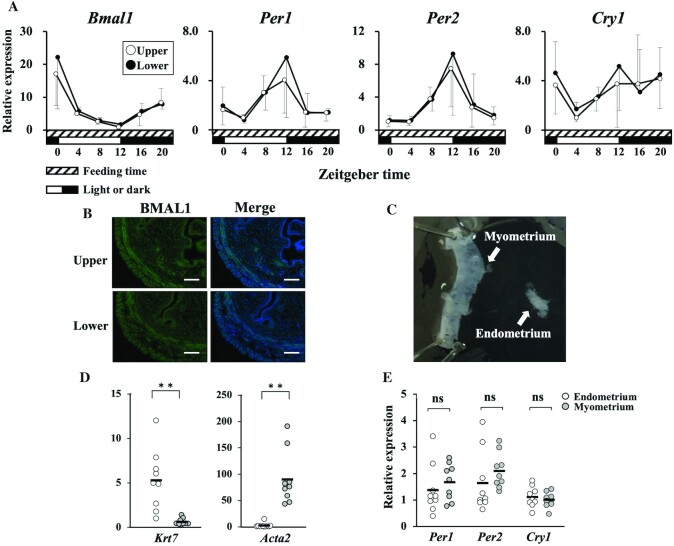
Synchronization of clock gene expression in the uterus. Uterine samples were obtained from 10-wk-old female mice (ad libitum consumption, *n *= 18) at 4-h intervals, ZT0, ZT4, ZT8, ZT12, and ZT16, and were divided into upper and lower parts for analysis of clock gene expression in each segment. (A) Both segments showed similar periodicity in the expression of clock genes (*Bmal1*, *Per1*, *Per2*, and *Cry1*) by qPCR. Relative expression is presented as fold units per minimal value of the upper segment. (B) Immunohistochemical analysis demonstrated no differences in the expression intensity of BMAL1 between the upper and lower segments of the uterus. We also observed no difference in *Bmal1* expression among the endometrium and inner and outer muscle layers. Bars show 100 µm. (C) The uterus additionally obtained from 10-wk-old female mice (ad libitum consumption, *n *= 10) at ZT12 was divided into endometrial and myometrial tissues. (D) The mRNA expression of *Krt7* and *Acta2* was significantly dominant in endometrial and myometrial tissues, respectively. **P < 0.01. (E) qPCR showed that there was no significant difference in the intensity of mRNA expression of clock genes (*Per1*, *Per2*, and *Cry1*) between endometrial and myometrial tissues. Differences in mRNA expression between the endometrium and myometrium were analyzed by the paired *t* test. *Acta2*, smooth muscle marker αSMA; *Bmal1*, brain and muscle aryl hydrocarbon receptor nuclear translocator-like protein 1; CK-7, cytokeratin 7; *Cry1*, cryptochrome 1; *Krt7*, endometrial epithelial marker CK-7; ns, nonsignificant; *Per*, period circadian clock; ZT, Zeitgeber time.

Using additional 10-wk-old female mice (ad libitum consumption, *n *= 18), uterine samples were obtained and divided into upper (ovarian side) and lower (vaginal side) parts, and the clock gene expression of each segment was analyzed by qPCR. Both upper and lower segments showed similar periodicity in clock gene expression (*Bmal1*, *Per1*, *Per2*, and *Cry1*) (**Figure 3**A).

In accordance with the mRNA expression of clock genes, immunohistochemical analysis revealed no differences in the expression intensity of BMAL1 protein between the upper and lower segments of the uterus (Figure 3B).

Because no differences in the expression of BMAL1 protein among the endometrium and inner and outer muscle layers were observed by immunohistochemical staining (Figure 3B), we examined the mRNA expression of other clock genes between endometrial and muscular tissues. Uterine samples were further obtained from 10-wk-old female mice (consuming ad libitum, *n *= 10) at ZT12 when the expression intensities of *Per1*, *Per2*, and *Cry1* were high (Figure 2A). The uterine tissues were divided into endometrial and myometrial parts (Figure 3C). The specificity of the separated tissues was confirmed by evaluating gene expression of the endometrial epithelial cell marker *Krt7* and smooth muscle marker *Acta2* (*P *< 0.01, Figure 3D). One sample with low specificity was omitted from further analysis. These qPCR results showed no significant difference in intensity of mRNA expression of clock genes (*Per1*, *Per2*, and *Cry1*) between endometrial and myometrial tissues (Figure 3E).

These findings suggest that the circadian rhythms of clock genes are synchronized throughout the uterine tissues.

### Feeding regulated circadian rhythms of uterine clock genes

**FIGURE 4:**
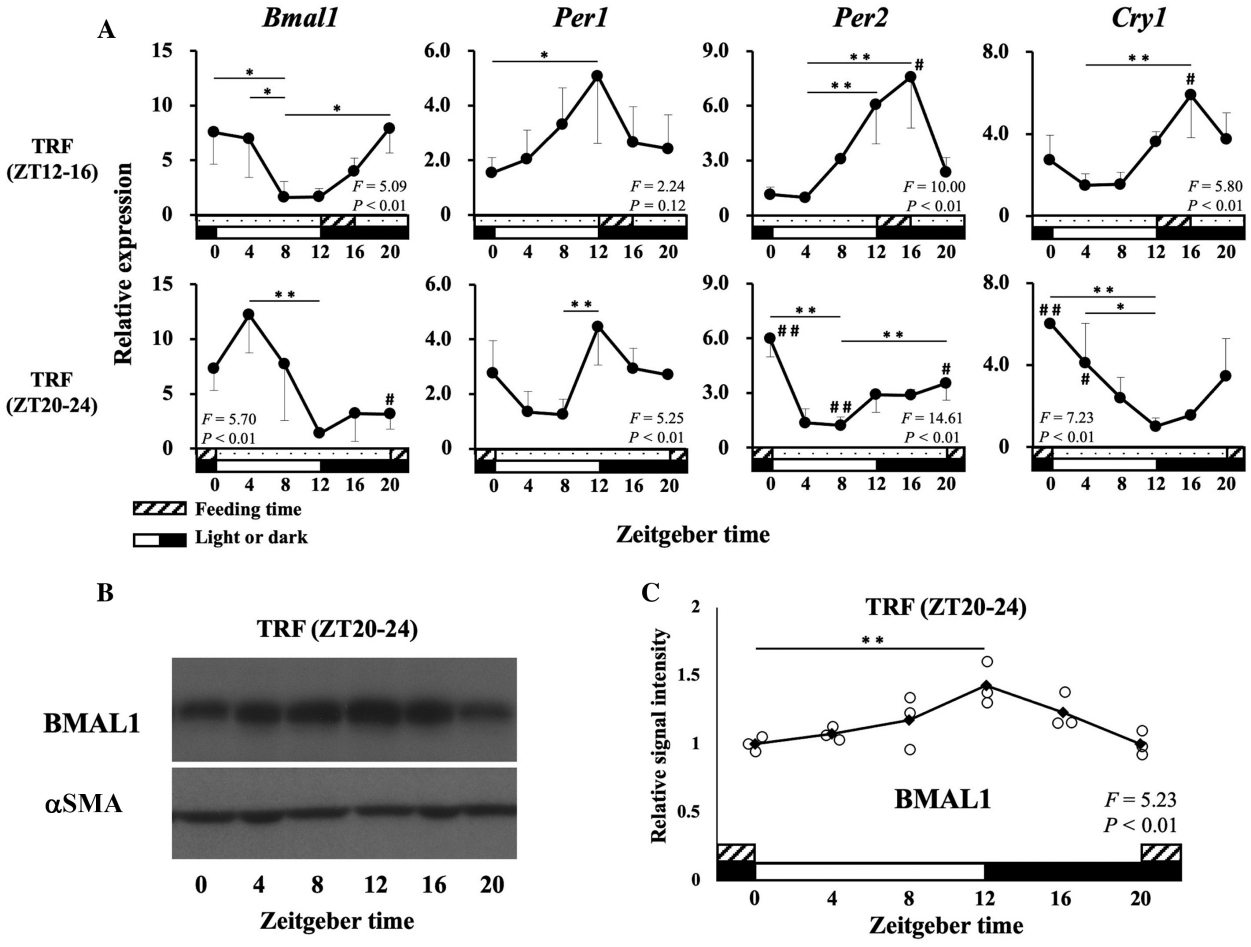
Circadian rhythms of uterine clock during time restriction of food intake. To examine the relation between the delay of feeding during the active phase and uterine circadian rhythms, the uterine clock gene expression profiles in groups II and III were analyzed by qPCR. (A) Consistent with the results in group I, *Bmal1* expression in group II (feeding during ZT12–16) was elevated after ZT12 at the start of the active phase and food intake. In contrast, *Bmal1* expression in group III (feeding during ZT20–24) was elevated after ZT20 at the start of food intake, showing a significantly backward shift of circadian expression. Relative expression is presented as fold units per minimal value. This 8-h shift in circadian rhythms was also observed in *Per2* and *Cry1*. (B, C) Western blot analysis confirmed a significant circadian rhythm of BMAL1 protein expression. The peak of protein expression was observed 8 h after that of mRNA expression. The cyclic changes in mRNA and protein expression of clock genes within each group (**P < 0.01; *P < 0.05, among ZTs) and the differences in mRNA expression of each ZT among groups I–III (^##^*P* < 0.01; ^#^*P* < 0.05, group II vs. group III) were analyzed by 1-factor ANOVA followed by the Dunnett test. αSMA, α-smooth muscle actin; *Bmal1*, brain and muscle aryl hydrocarbon receptor nuclear translocator-like protein 1; *Cry1*, cryptochrome 1; *Per*, period circadian clock; TRF, time restriction of food intake; ZT, Zeitgeber time.

Consistent with the results in group I, *Bmal1* expression in group II was elevated after ZT12 at the start of the active phase and food intake (**Figure 4**A). In contrast, *Bmal1* expression in group III was elevated after ZT20 at the start of food intake (Figure 4A). This 8-h shift in circadian rhythms was also observed in other clock genes (*Per2* and *Cry1*) (Figure 4A). Western blot analysis confirmed a significant circadian expression of BMAL1 protein, and the peak of its expression was observed 8 h after that of mRNA expression (Figure 4B and C).

These findings indicate that the circadian rhythm of the uterine clock can be shifted according to the first meal occasion in the active phase.

## Discussion

Mammals have evolved the uterus, an organ specialized for reproduction, which undergoes embryo implantation and achieves fetal delivery. This study showed that the murine uterus has circadian rhythms of clock gene expression under ad libitum food consumption (group I). These results suggest that the circadian rhythm of *Bmal1* is regulated by the light/dark cycle. However, considering that eating activity becomes marked at the beginning of the dark period (active phase), it is also possible that *Bmal1* mRNA expression is associated with the timing of food intake. In rodents and humans, it is widely accepted that peripheral circadian oscillators operate in various organs under the molecular cascades of clock gene products (22, 23). The rat uterus was reported to show circadian rhythms of clock genes (17, 24). Because the rhythmic patterns observed in rats are similar to those of this study, it is suggested that common mechanisms regulate the uterine peripheral clock in rodents.

This study also showed that the circadian expression of *Bmal1*, a leading gene of molecular cascades during the circadian rhythm, is reset by dietary intake, inducing upregulation of mRNA expression of *Bmal1*. This suggests that the uterine peripheral clock is more strongly regulated by diet than by the light/dark cycle, which is a main regulator of the central clock (25, 26). To our knowledge, this is the first report that the first meal occasion in the active phase regulates a circadian rhythm of the uterine peripheral clock. In general, peripheral oscillators in most organs are mainly regulated by daily food intake cycles (27). In the liver, a hepatic circadian clock mediates the circadian regulation of hepatic glucose production, whereas dietary intake regulates a rhythm of the hepatic circadian clock (28). In contrast to these organs that are vital for survival, the physiological significance of dietary regulation of the uterine peripheral clock is unclear. However, when pregnant, the fetus cannot receive light stimulation in the intrauterine environment. Consequently, transplacental glucose transport after maternal food intake is one of the direct circadian signals for the fetus (29). The presence of clock gene oscillation was reported in the uterus, placenta, and fetal membranes of pregnant mice (30). Accordingly, it is reasonable to speculate that the uterus synchronizes its function with food intake to prepare an adequate environment for the fetus.

Because both food intake and the light/dark cycle are the main regulators of circadian rhythms (25, 26), we consider that skipping breakfast at the start of the active phase interferes with the central clock system (31) and this disruption of the circadian rhythm by meal skipping affects reproductive rhythms, leading to ovarian and uterine dysfunction (9, 11). Consistent with this, we previously observed that ovulations in daytime-fed young female rats were significantly impaired, indicating that the timing of food intake is an important factor regulating the hypothalamic-pituitary-ovarian axis (11). Considering additional new information that the timing of food intake can regulate the circadian rhythm of the uterine clock, we hypothesize a novel mechanism whereby meal skipping directly disrupts the circadian rhythm of the uterine clock system, leading to uterine dysfunction (32).

In addition to breakfast skipping, we previously reported that female college students with a history of dieting showed a significant increase in dysmenorrhea and menstrual problems (8, 33). Importantly, the intensity of dysmenorrhea in students currently on a diet is lower than that of students who have never dieted (33). We speculate that the common problem between breakfast skipping and dieting is starvation stress, because meal skipping at the start of the active phase by breakfast skipping extends the starvation period during the active phase, whereas reduction of food consumption by dieting enhances the intensity of starvation stress. Furthermore, we found that patients with a history of dysmenorrhea around the age of 20 y have a significantly higher risk of developing hypertensive disorders of pregnancy in adulthood (34). As a pathological sign of uterine functions, dysmenorrhea is one of the important gynecological symptoms, considered to be caused by abnormal myometrial contraction (35). Collectively, we propose that inadequate dietary habits during adolescence and young adulthood inhibit the development and maturation of reproductive organs; this is memorized and leads to the latent progression of reproductive dysfunction and later onset of obstetrical and gynecological diseases. We termed this concept “adolescent dietary habit–induced obstetric and gynecologic disease” (ADHOGD) (10). Although we did not evaluate the changes in uterine functions in breakfast skipping model mice (group III), this study provides new evidence to explain the mechanism of ADHOGD, proposing that inadequate dietary habits impair uterine functions through a uterine clock system.

In the present study, we observed that clock gene expression is synchronized throughout the uterus from longitudinal and vertical axes. To our knowledge, this is the first report to describe the synchronization of circadian rhythms of clock gene expression among uterine components. The uterus concentrically consists of functionally different elements: endometrium and myometrium. The endometrium provides a specific immunological and tissue remodeling environment to facilitate embryo implantation (32, 36–38), whereas the myometrium generates sophisticated and dynamic peristaltic motions to control embryo implantation and delivery of the fetus (39, 40). In mice, the myometrium is divided into inner circular and outer longitudinal muscle layers. The coordinated contractions between inner and outer layers can produce fine-tuned uterine peristaltic movements, which were reported to contribute to embryo spacing on the longitudinal axis and embryo orientation on the vertical axis (41). Recently, we identified a novel middle layer of the myometrium using a tissue-clearing method (42). This new layer anatomically connects the outer longitudinal and inner circular muscles and contains pacemaking telocytes (43), suggesting that this middle layer is a regulatory center that coordinates uterine contractions. Consequently, the periodic synchronization of clock gene expression throughout the uterus suggests that the uterine clock system plays an important role in the coordinated uterine functions. Using transgenic mice, it was reported that delivery rates during the normal period were reduced in myometrium-specific *Bmal1*-knockout mice (44). More importantly, our preliminary experiments showed that uterus-specific *Bmal1*-knockout mice failed to maintain pregnancy (M Ono, et al., unpublished results, 2021). These findings strongly support the physiological contribution of a peripheral clock system to uterine functions.

This study has several limitations. First, we did not evaluate changes in the amount of food consumed, body weight, or energy consumption in the time-restricted mouse models. Previously, we observed that breakfast skippers with dysmenorrhea showed no decrease of BMI, and the intensity of dysmenorrhea in students currently dieting is lower than that of students who have never dieted in Japanese female college students. However, the food restriction protocols in this study might have altered catabolic stress conditions in mice. Because meal skipping was reported to be associated with lower energy consumption (45), we should note the possibility that reduced energy consumption due to breakfast skipping causes alterations in the uterine circadian cycle and dysmenorrhea. Second, we did not evaluate the effects of ovarian steroid hormones on uterine clock gene expression. Estrogen was previously shown to affect a circadian rhythm of the uterine clock under the condition of constant darkness in rats (46). Later, the authors reported the possibility that a circadian rhythm of the rat uterine clock slightly changes within 4 h during the estrus cycle, where the peak of *Per2* mRNA expression was gradually delayed from ZT12 in proestrus to ZT16 in diestrus (24). Although this shift was not detected in *Bmal1* and *Per1*, they further observed that estrogen and progesterone can change the rhythm and amplitude of *Per2* expression ex vivo using primary uterine explants, proposing that circulating ovarian steroid hormones modulate oscillators in sex hormone–sensitive organs during the estrus cycle (24). In contrast to ovarian hormone–induced modulation, this study demonstrated a distinct 8-h shift of circadian rhythms of the uterine clock, which was adjusted to the timing of food intake. Although the present study did not examine the influence of the ovarian function, this shift cannot be explained by the hormonal changes of the ovary alone.

In conclusion, this study indicates that time-restricted feeding regulates a circadian rhythm of the murine uterine clock that is synchronized throughout the uterine body. These findings suggest that the uterine clock system is a new candidate to explain the etiology of breakfast skipping–induced uterine dysfunction.

## Notes

This work was supported in part by Grant-in-Aids for Scientific Research (nos. 17H04337, 18K08470, 19H01617, and 19K22681), a Health and Labor Sciences Research Grant (no. 19216689), and the Japan Agency for Medical Research and Development (nos. 19gk0110024h0002, 19gk0210016h0001, 19cm0106610h0002, and 20ck0106549h0001) from the Japanese government.

Author disclosures: The authors report no conflicts of interest.

TH and MO contributed equally to this study.

Abbreviations used: *Acta2*, smooth muscle marker αSMA; ADHOGD, adolescent dietary habit–induced obstetric and gynecologic disease; BMAL1, brain and muscle aryl hydrocarbon receptor nuclear translocator-like protein 1; CK-7, cytokeratin 7; CLOCK, clock circadian regulator; CRY, cryptochrome; *Krt7*, endometrial epithelial marker CK-7; *Per*, period circadian clock; PFA, paraformaldehyde; ZT, Zeitgeber time; αSMA, α smooth muscle actin.
